# Dragonfly Algorithm and Its Hybrids: A Survey on Performance, Objectives and Applications

**DOI:** 10.3390/s21227542

**Published:** 2021-11-13

**Authors:** Bibi Aamirah Shafaa Emambocus, Muhammed Basheer Jasser, Aida Mustapha, Angela Amphawan

**Affiliations:** 1 Department of Computing and Information Systems, School of Engineering and Technology, Sunway University, Petaling Jaya 47500, Selangor, Malaysia; 17037730@imail.sunway.edu.my (B.A.S.E.); angelaa@sunway.edu.my (A.A.); 2 Department of Mathematics and Statistics, Faculty of Applied Sciences and Technology, Universiti Tun Hussein Onn Malaysia, Batu Pahat 86400, Malaysia, Johor; aidam@uthm.edu.my

**Keywords:** dragonfly algorithm, swarm intelligence, optimization

## Abstract

Swarm intelligence is a discipline which makes use of a number of agents for solving optimization problems by producing low cost, fast and robust solutions. The dragonfly algorithm (DA), a recently proposed swarm intelligence algorithm, is inspired by the dynamic and static swarming behaviors of dragonflies, and it has been found to have a higher performance in comparison to other swarm intelligence and evolutionary algorithms in numerous applications. There are only a few surveys about the dragonfly algorithm, and we have found that they are limited in certain aspects. Hence, in this paper, we present a more comprehensive survey about DA, its applications in various domains, and its performance as compared to other swarm intelligence algorithms. We also analyze the hybrids of DA, the methods they employ to enhance the original DA, their performance as compared to the original DA, and their limitations. Moreover, we categorize the hybrids of DA according to the type of problem that they have been applied to, their objectives, and the methods that they utilize.

## 1. Introduction

Optimization algorithms are essential for numerous optimization applications where usually certain parameters are minimized or maximized by considering an objective function. Optimization algorithms can be classified as either deterministic or non-deterministic [1]. Deterministic algorithms are exact methods, and usually they need a substantial amount of time and resources for solving large optimization problems. Hence, non-deterministic algorithms, also called heuristic algorithms, are being increasingly used and developed. They can be based on various natural processes; for example, trajectory-based, physics-based or population-based, which can be either nature- or bio-inspired [1]. Swarm intelligence algorithms are classified as nature-inspired population-based heuristic optimization algorithms.

Swarm intelligence is a discipline which is utilized for solving optimization problems by producing low cost, fast and robust solutions. Its technique consists of making use of a number of agents, thereby forming a population in which individuals interact among themselves and with their environment, to give rise to a global intelligent behavior. There exist numerous swarm intelligence algorithms, such as ant colony optimization (ACO), grey wolf optimization (GWO), firefly algorithm (FA), whale optimization algorithm (WOA), bee colony optimization (BCO), and particle swarm optimization (PSO).

The Dragonfly Algorithm (DA) is a swarm intelligence algorithm that was proposed in 2016 [2], and it is inspired by the behavior of dragonflies in nature. It has been found to have a higher performance than some of the most popular evolutionary algorithms, such as the genetic algorithm (GA), and swarm intelligence algorithms such as particle swarm optimization (PSO). Owing to its high effectiveness and efficiency, it has been utilized in multifarious applications and attempts to further improve its performance have been made and hence a number of hybrids of DA have been proposed. Our motivation for working on this algorithm is that DA and its hybrids have proven to be useful in multifarious applications and they also have a higher performance as compared to other swarm intelligence algorithms and their hybrids.

We have found that there are insufficient studies relating to the applications of the dragonfly algorithm and its hybrids. Although there are some surveys about the dragonfly algorithm, we have identified certain limitations relating to the surveys on DA. The hybrids of DA have never been categorized according to the type of problem that they have been applied to, whether continuous and single-objective problems, binary and single-objective problems, or continuous and multi-objective problems. The different versions of the hybrid algorithms, that is, the continuous, binary or multi-objective versions have not been considered. There are no taxonomies which categorize the hybrids of DA according to their objective, that is whether they improve the effectiveness or efficiency of the original DA or both. Moreover, all the hybrids that have been proposed focus on improving the effectiveness of the original DA; however, there are no taxonomies which cluster the hybrids based on the method employed to improve the effectiveness. In this paper, we present a more comprehensive survey on the dragonfly algorithm by covering the aforementioned limitations.

Table 1 shows a comparison between our survey and the previous surveys on DA in terms of the contents presented in the surveys.

The remainder of the paper is structured as follows: in Section 2, a background on the dragonfly algorithm is presented, in Section 3, an explanation on the hybrids of DA is presented, followed by a discussion on the applications of both DA and its hybrids in Section 4. In Section 5, a discussion on some challenges and future directions is given and finally in Section 6, the conclusions and future works are presented.

## 2. Dragonfly Algorithm

**Figure 1:**
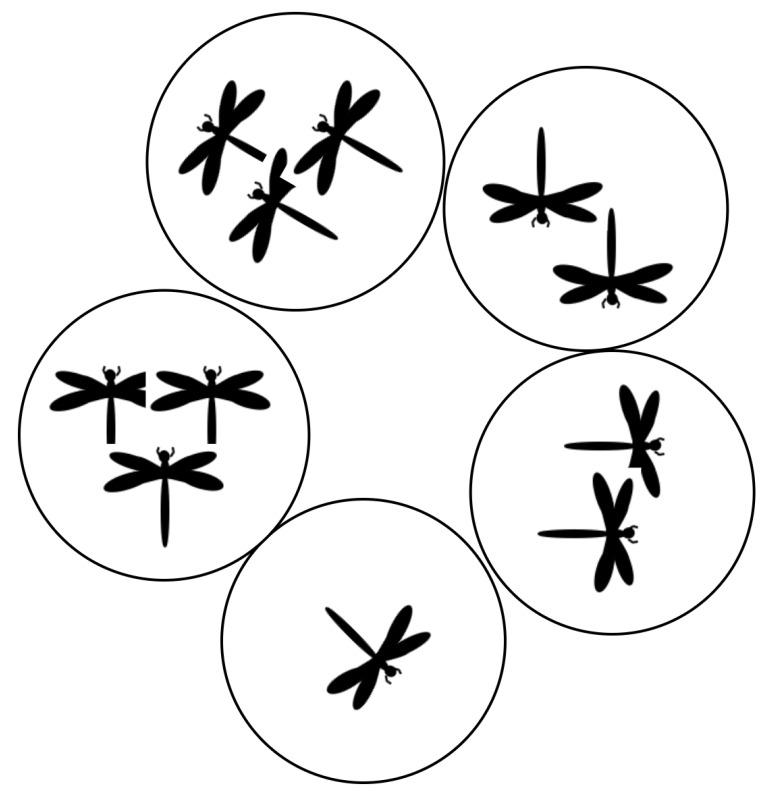
Static Swarm [2,5,6].

**Figure 2:**
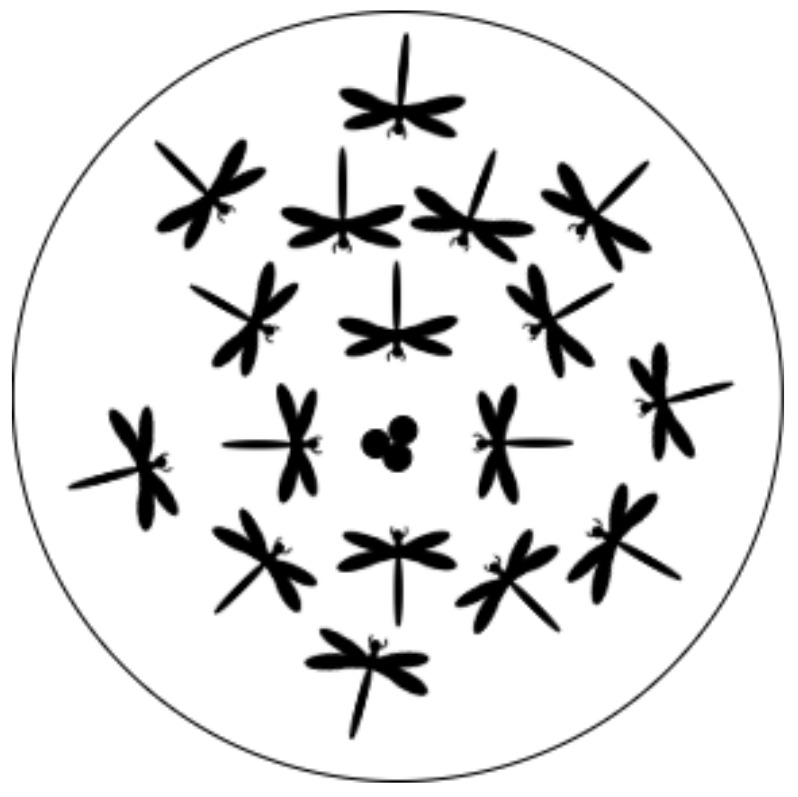
Dynamic Swarm [2,5,6].

The inspiration for the dragonfly algorithm is derived from the static and dynamic swarming behaviours of dragonflies in nature. The static and dynamic swarming behaviors are representative of the two requisite phases of optimization: exploration and exploitation. In a static swarm, as in Figure 1, dragonflies create sub-swarms and fly over different regions. This is tantamount to exploration, and it helps the algorithm to locate good areas of the search space. Conversely, in a dynamic swarm, as in Figure 2, dragonflies fly in a bigger swarm and along the same direction. This type of swarming is equivalent to the exploitation of an algorithm, which helps it to converge to the global optimum.

Five factors are used for to direct the dragonflies in the exploration and exploitation phases; namely, separation, alignment, cohesion, food factor and enemy factor. The separation weight (s), alignment weight (a), cohesion weight (c), food factor (f), enemy factor (e) and the inertia weight (w) are used for controlling the factors. The goal is to ensure that the swarm survives by attracting it towards food sources and distracting it away from enemies. The best solution found in an iteration is selected as the food source and the worst solution found is selected as the enemy. The weights of the factors are adjusted so as to have high alignment and low cohesion in the exploration phase and low alignment and high cohesion in the exploitation phase. The weights are changed accordingly to allow the transition of the algorithm from the exploration to the exploitation phase.

The separation factor is used to avoid the static collision of one dragonfly from other dragonflies in the neighborhood, and it is calculated as follows:

(1) $$\;S_{i}\;=\;-\;\sum_{j=1}^{N}X_{i}-X_{j}\;$$

where $X_{i}$ is the position of the current dragonfly, $X_{j}$ is the position of the *j*-th neighbour and *N* is the number of neighbouring dragonflies.

The alignment factor is used to match the velocity of one dragonfly to that of other dragonflies in the neighborhood and it is calculated as follows:

(2) $$A_{i}\;=\;\;\frac{\sum_{j=1}^{N}V_{j}}{N}$$

where $V_{j}$ is the velocity of the *j*-th neighbour and *N* is the number of neighbouring dragonflies.

The cohesion factor is the tendency of one dragonfly towards the center of mass of the neighborhood and is calculated as follows:

(3) $$C_{i}\;=\;\;\frac{\sum_{j=1}^{N}X_{j}}{N}\;-\;X_{i}\;$$

where $X_{j}$ is the position of the *j*-th neighbour, and *N* is the number of neighbouring dragonflies.

The food factor is the attraction of a dragonfly towards a food source and is calculated as follows:

(4) $$F_{i}\;=\;\;X^{+}\;-\;X_{i}$$

where $X^{+}$ is the position of the food source.

The enemy factor is the distraction of a dragonfly from an enemy, and it is calculated as follows:

(5) $$E_{i}\;=\;\;X^{-}\;+\;X_{i}$$

where $X^{-}$ is the position of the enemy.

Two vectors, a step vector (ΔX) and a position vector (*X*), are used to simulate movements and to update the position of the artificial dragonflies in a search space. The step vector is defined as:

(6) $$\Delta X_{i}^{t+1}=\left(sS_{i}+aA_{i}+cC_{i}+fF_{i}+eE_{i}\right)+w\Delta X_{i}^{t}$$

where *s* is the separation weight, $S_{i}$ is the separation of the *i*-th dragonfly, *a* is the alignment weight, $A_{i}$ is the alignment of *i*-th dragonfly, *c* is the cohesion weight, $C_{i}$ is the cohesion of the *i*-th dragonfly, *f* is the weight of the food factor, $F_{i}$ is the food factor of the *i*-th dragonfly, *e* is the weight of the enemy factor, $E_{i}$ is the enemy factor of the *i*-th dragonfly, *w* is the inertia weight, and *t* is the iteration counter.

After calculating the step vector, the position of the dragonflies is updated using:

(7) $$X_{i}^{t+1}=X_{i}^{t}+\Delta X_{i}^{t+1}$$

The neighbours of each artificial dragonfly are considered by assuming a radius around each one of them. To transition from the exploration to the exploitation phase, the radius of the neighbourhoods is incremented proportionally to the iteration counter so that the static swarms are changed to dynamic swarms. During the last stage of optimization, all the dragonflies will come together to form one dynamic swarm which will converge towards the global optimum solution. The Lévy flight mechanism [7] is used for the artificial dragonflies to navigate around the search space when they have no neighbours. It is a random walk which is used to generate a random position for dragonflies which have no neighbours. In this case, the position update formula used is:

(8) $$X_{i}^{t+1}=X_{i}^{t}+Levy\left(d\right)\times X_{i}^{t}$$

where *t* is the current iteration number and *d* is the dimension of the position vectors.

The step vector and position vectors of each dragonfly are updated in every iteration until the end criterion is met. The pseudocode of the dragonfly algorithm is given in Algorithm 1.
**Algorithm 1:** Dragonfly Algorithm
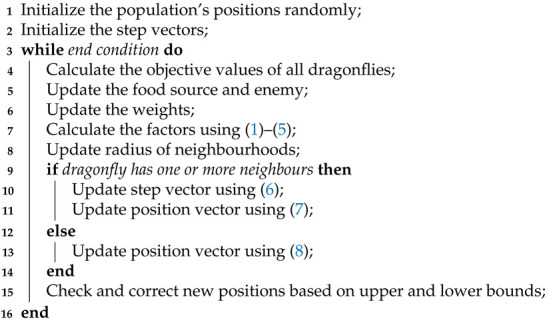


## 3. Hybrids of DA

The original DA algorithm is apt to be used for continuous and single objective optimization problems. A continuous problem means that a potential solution for the problem can have any real value within a specified range and single-objective means that the problem has only one objective; this is either to minimize or maximize a single objective function. In [2], along with the original dragonfly algorithm, the binary dragonfly called BDA and the multi-objective algorithm called MODA are also proposed. The BDA algorithm can be applied to binary or discrete single-objective optimization problems and the MODA algorithm can be applied to continuous and multi-objective optimization problems. A binary or discrete problem means that there is a finite set of potential solutions for the problem. A multi-objective problem means that the problem consists of more than one objective, and hence, there is more than one objective functions to be minimized or maximized.

**Figure 3:**
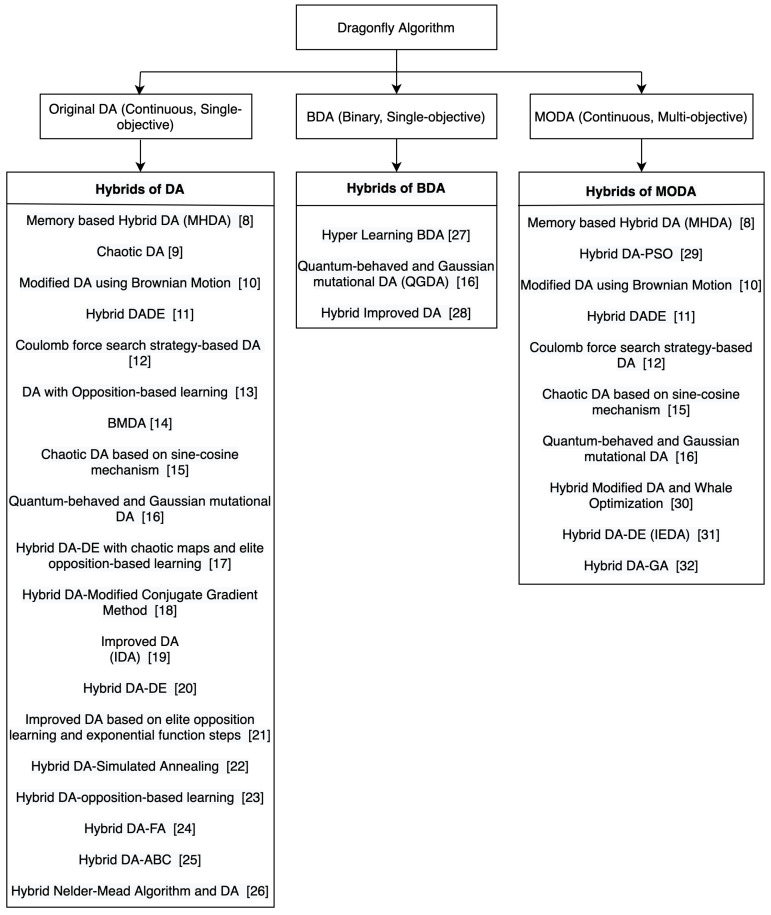
Taxonomy categorizing hybrids of DA.

**Figure 4:**
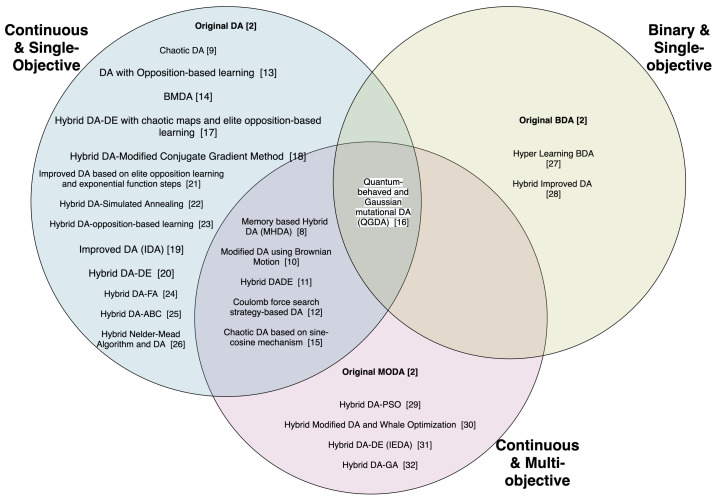
Taxonomy categorizing hybrids of DA.

We identified the variants and hybrids of DA which have been applied to continuous, binary and multi-objective problems and categorized them in terms of those which have been applied to continuous and single-objective problems, binary or discrete and single-objective problems and continuous and multi-objective problems. The taxonomies in Figure 3 and Figure 4 show this categorization of the variants and hybrids of DA.

In Figure 3, the taxonomy shows the categorization of the hybrids of DA according to the type of problems to which they have been applied. While most of the proposed hybrids have been applied to only one type of problem, some of them have been applied to more than one type of problem, and hence, they appear more than once in the taxonomy.

In the taxonomy in Figure 4, the intersection between ’Continuous and Single-Objective’ and ’Continuous and Multi-objective’ shows that there are two versions of the hybrid algorithms, one which has been applied to a continuous and single-objective problem, and one applied to a continuous and multi-objective problem. The intersection of the three sets indicates that there are three versions of the hybrid algorithm, one which has been applied to a continuous and single-objective problem, one applied to a continuous and multi-objective problem, and one applied to a binary and single-objective problem. As for the non-intersecting parts, it means that there is only one version of the hybrid algorithm which has been applied to either a continuous and single-objective problem, a binary and single-objective problem, or a continuous and multi-objective problem.

### 3.1. Hybrids of DA Which Handle Continuous and Single-Objective Problems

In this section, we discuss the hybrids of the dragonfly algorithm which have been applied for solving continuous and single-objective problems, and hence there exists the continuous version of the algorithm. Some of these algorithms have also been used for binary or multi-objective problems, and therefore the binary and single-objective or the continuous and multi-objective versions of these algorithms have also been proposed in addition to the continuous and single-objective one.

In [8], the memory-based hybrid DA (MHDA) is proposed, which caters for continuous and single-objective problems. It has been tested using the CEC 2014 benchmark functions, which are continuous numerical optimization problems. They are also single-objective, since there is only one objective function to be optimized. Hence, a continuous and single-objective version of the algorithm is proposed. This algorithm also has a continuous and multi-objective version.

In [9], a chaotic DA is proposed to be applied in feature selection. It is used to minimize the size of the selected features, which will in turn maximize the classification performance and decrease the classification computational cost. Since the problem has only one objective; that is, to minimize the size of the selected features, and the size can be any real value within a certain range, a continuous and single-objective version of the algorithm is proposed.

In [10], a modified DA algorithm using Brownian motion is proposed. It has been tested using single-objective and multi-objective benchmark functions. Hence, two versions of the algorithm are proposed; one which caters for continuous and single-objective problems and one for continuous and multi-objective problems.

In [11], a hybrid DADE algorithm which is a hybrid of differential evolution and DA is proposed. The algorithm has been tested using benchmark mathematical functions which are single-objective and continuous. Hence, a continuous and single-objective version of the algorithm is proposed. A multi-objective version of this hybrid algorithm is also proposed.

A coulomb force search strategy-based DA is proposed in [12]. A continuous and single-objective version and a continuous and multi-objective version of the algorithm have been proposed.

In [13], DA with opposition-based learning (OBL), called OBLDA is proposed. It is used in multilevel thresholding colour image segmentation to find the optimal threshold value for each colour component. Since the problem has a single-objective, which is to find the optimal threshold value, and that value can be any real value within a specified range, a continuous and single-objective version of the algorithm is proposed.

In [14], a biogeography-based and Mexican hat wavelet DA (BMDA) is proposed. The algorithm is tested using benchmark functions from the CEC2017 library. These functions are continuous numerical optimization problems which are single-objective, and hence a continuous and single-objective version of the proposed algorithm is used.

A chaotic DA based on sine-cosine mechanism (SC-DA) is proposed in [15]. The algorithm is examined using numerical benchmark functions which are continuous and single-objective, and hence a continuous and single-objective version of the algorithm is proposed.

A quantum-behaved and Gaussian mutational DA (QGDA) is proposed in [16]. It is validated using CEC 2014 benchmark functions, and it is applied in feature selection to obtain an optimal subset of features. Hence, a continuous and single-objective version of the proposed algorithm is used. A continuous and multi-objective version of this algorithm is also proposed.

A hybrid DA-DE algorithm with chaotic maps and elite opposition-based learning (EOBL) is proposed in [17]. The algorithm is used in multilevel thresholding image segmentation to obtain the optimal threshold values. This problem has a single-objective, which is to find the optimal threshold value, and that value can be any real value within a certain range. Therefore, a continuous and single-objective version of the algorithm is proposed.

A hybrid DA-modified conjugate gradient method is proposed in [18]. The algorithm is tested using standard functions for single-objective numerical optimization, and hence a continuous and single-objective version of the proposed algorithm is proposed.

In [19], an improved DA, called IDA is proposed. The IDA algorithm has been applied to optimize the parameters of a Support Vector Machine (SVM). Since the problem is single-objective, that is, to optimize the parameters of the SVM and the parameters can have continuous values, a continuous and single-objective version of the algorithm is proposed.

A hybrid DA-DE is proposed in [20] to be applied to image segmentation, so as to determine the optimal threshold values. This is a continuous problem, as the threshold values can have any real value within a certain range and also single-objective problem, as there is only one objective function. Hence, a continuous and single-objective version of the algorithm is proposed.

In [21], an improved DA based on elite opposition learning and exponential function steps, called EOEDA is proposed. The proposed algorithm is tested using numerical optimization to find the optimal values for single-objective standard functions, and hence a continuous and single-objective version of the algorithm is proposed.

In [22], a hybrid DA-Simulated Annealing algorithm is proposed. The algorithm is applied to the flexible flow-shop scheduling to optimize the online scheduling sequence. A continuous and single-objective version of the algorithm is proposed.

In [23], a hybrid DA-opposition-based learning algorithm is proposed. The algorithm is tested on real parameter function optimization to obtain the optimal parameters for the functions. It is a continuous problem, since the parameters can have any real value within a specified range, and single-objective, since it consists of only one objective function. Hence, a continuous and single-objective version of the algorithm is proposed.

In [24], a hybrid of DA and firefly algorithm called DA-FA is proposed. It is applied to the wireless sensor networks localization problem to locate the position of nodes using the position of an anchor node, and a continuous and single-objective version of the algorithm is used.

In [25], a hybrid of DA and artificial bee colony (ABC) called DA-ABC is proposed. It is used to optimize the weights of a multilayer perceptron (MLP) during its training process. Since the problem is single-objective; that is, to optimize the weights of the MLP, and the weights can be any real value within a certain range, a continuous and single-objective version of the algorithm is proposed.

In [26], a hybrid DA and Nelder–Mead algorithm called INMDA is proposed, which is used to train a multilayer perceptron to determine the most optimal weights and biases for the MLP. Hence, a continuous and single-objective version of the algorithm is proposed.

### 3.2. Hybrids of DA Which Handle Binary and Single-Objective Problems

Other hybrids of the dragonfly algorithm have been applied to binary or discrete optimization problems. These problems are usually single-objective problems. The algorithms discussed in this section, specifically the ones which have been proposed in [27,28], have only been applied to binary and single-objective problems and hence only the binary version of these algorithms have been proposed.

In [27], a hyper learning binary dragonfly algorithm (HLBDA) is proposed to be applied to feature selection to determine the optimal subset of features for a classification problem. This is a single-objective problem which is to find an optimal subset of features, and it is also a discrete problem, since each potential solution is a subset of features, and there are a limited number of subsets which can be considered. Hence, a binary and single-objective version of the HLBDA algorithm is proposed and applied to the problem.

In [28], a hybrid improved DA is proposed to be applied to feature selection to determine the optimal subset of features. Since this is a discrete problem with a single-objective, a binary and single-objective version of the algorithm is proposed.

The quantum-behaved and Gaussian mutational DA (QGDA) [16], which has been discussed in the previous section, has also been applied to a binary problem, and hence a binary and single-objective version of the algorithm has also been proposed. The binary and single-objective version of the algorithm is applied to feature selection to determine the optimal subset of features.

### 3.3. Hybrids of DA Which Handle Continuous and Multi-Objective Problems

In this section, the hybrids of DA which have been applied to continuous and multi-objective problems are discussed. Some of these algorithms, specifically the ones proposed in [29,30,31,32], have only been applied to continuous and multi-objective problems, and hence only the multi-objective version of the algorithms has been proposed.

In [29], a hybrid DA-PSO algorithm is proposed to solve the multiobjective optimal power flow (MO-OPF) problem to minimize the fuel cost, emissions, and transmission losses, while satisfying some equality and inequality constraints. This problem has more than one objective, and these are: to minimize the fuel cost, emissions, and transmission losses, and at the same time, to satisfy some equality and inequality constraints. It is also a continuous problem, since the parameters can have any real value within a certain range. Hence, a continuous and multi-objective version of the algorithm is proposed and applied to the problem.

In [30], a hybrid modified DA and whale optimization is proposed to optimally schedule microgrid with islanding constraints to achieve the best quality. Since this is a multi-objective problem where the parameters can have any real value within a specified range, the continuous and multi-objective version of the algorithm is proposed and used.

In [31], a hybrid DA-DE, called IEDA, is proposed for the optimal design of a hybrid power active filter. Since this is a multi-objective problem where the parameters can have any real value within a specified range, the continuous and multi-objective version of the algorithm is proposed and used.

In [32], a hybrid of DA and genetic algorithm (GA), called DA-GA, is proposed to solve the optimal power flow problem. This is also a continuous and multi-objective problem, and hence the continuous and multi-objective version of the algorithm is proposed and applied.

The MHDA algorithm [8], modified DA using Brownian motion [10], hybrid DADE [11], Coulomb force search strategy-based DA [12], SC-DA [15] and QGDA [16], which have been discussed in the earlier sections, have also been applied to multi-objective problems, and continuous and multi-objective versions of these algorithms have also been proposed. The continuous and multi-objective version of the MHDA algorithm [8], modified DA using Brownian motion [10], hybrid DADE [11] and QGDA [16] have been used for the optimal design of a welded beam. The Coulomb force search strategy-based DA [12] has been applied for the optimal design of a bucket wheel reclaimer (BWR), and the SC-DA [15] has been applied for the optimal design of a cantilever beam.

### 3.4. Performance Analysis

While most of the algorithms increase the performance of the original DA in terms of effectiveness, that is enhancing the quality of solutions, some algorithms also improve its performance in terms of efficiency; that is, increasing the convergence rate. In order to achieve these objectives, different methods are employed. We identified four main techniques by which the effectiveness of the original dragonfly algorithm is enhanced: by improving the exploitation of DA, that is the local search, by improving the exploration which is the global search of DA, by improving both exploitation and exploration or by improving the initialization; that is, finding better initial positions for the artificial dragonflies.

**Figure 5:**
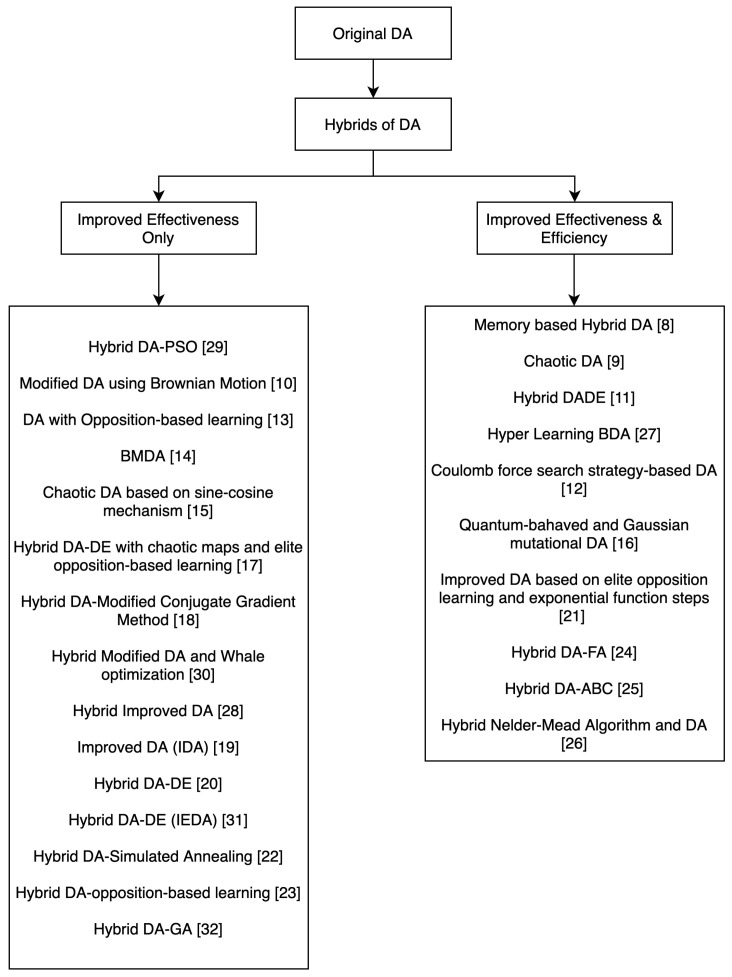
Taxonomy categorizing hybrids of DA in terms of improved effectiveness and efficiency.

The taxonomy in Figure 5 shows the existing hybrids of DA categorized into whether they improve only the effectiveness of the original DA or both the effectiveness and efficiency.

**Figure 6:**
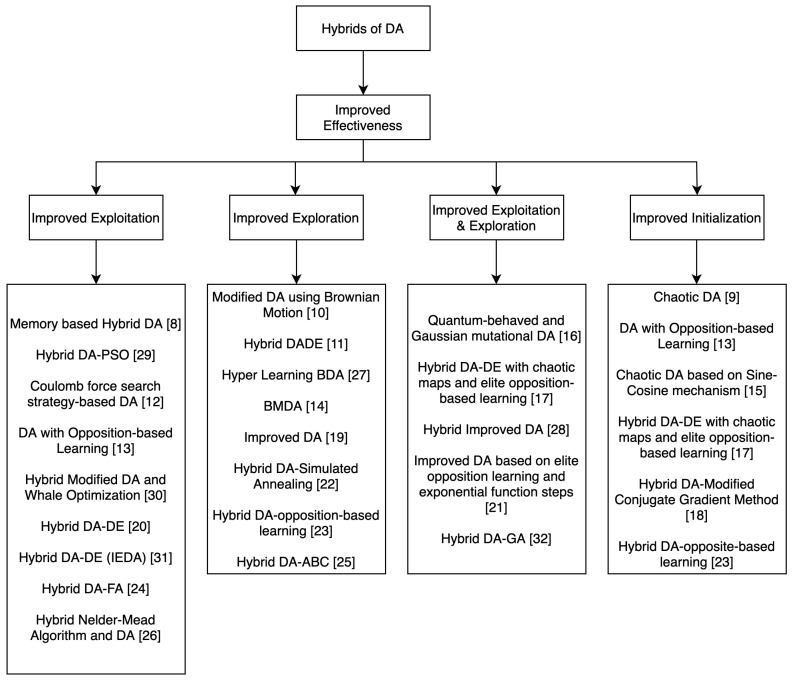
Taxonomy categorizing hybrids of DA in terms of effectiveness improving method.

The taxonomy in Figure 6 categorizes the hybrids of DA in terms of the technique employed to improve its effectiveness.

#### 3.4.1. Hybrids of DA Which Improve Its Effectiveness by Improving Exploitation

Table 2 shows the hybrids of DA which have improved the effectiveness of the original DA by means of improving its exploitation phase in terms of the algorithm used to improve DA, the way of improvement, the application that they have been used for, how much the effectiveness of DA is improved, and whether the efficiency is also improved or not. In the ’Improved effectiveness (%)’ column, the problem for which this improvement is achieved is shown within parenthesis. In the ’way of improvement’ column, the phase and the equations or steps which are improved are shown.

The hybrid algorithms MHDA [8], Coulomb force search strategy-based DA [12], DA-FA [24] and INMDA [26] improve the effectiveness of DA by improving its exploitation phase, and these hybrids are also able to improve the efficiency of DA; that is, increasing its convergence rate. Some detailed explanations about these algorithms which are not presented in Table 2 are given next.

The memory-based hybrid DA (MHDA) [8] improves the effectiveness of DA by overcoming the low exploitation problem, which causes the algorithm to prematurely converge to local optima. This is done by hybridizing the DA with PSO and by adding a memory element in DA. The memory element is used to keep track of the best solution found by each dragonfly of DA and the best solution found in each neighborhood of dragonflies. The dragonfly algorithm with internal memory first converges the search space to good regions. The PSO algorithm is then initialized using the matrix of the best solutions obtained by DA for further exploitation. Hence, the algorithm employs the exploration capability of DA and exploitation of PSO. The exploitation of DA is improved; since once a dragonfly updates its position, PSO is initialized with its personal best solution and global best solution of the neighbourhood so as to further exploit the search area and to find a better position. Two equations of PSO, namely, a modified velocity update and the position update equations, are added to the DA algorithm, as shown in Table 2. The parameters $C_{1}$ and $C_{2}$ are the cognitive and social parameters, $r_{1}$ and $r_{2}$ are random numbers between 0 and 1. $DA-pbest_{i}^{t}$ and $DA-gbest_{i}^{t}$ are the personal and global best particles of PSO, respectively.

In [12], a Coulomb force search strategy-based DA is proposed to improve the effectiveness of DA by enhancing its exploitation capability, thereby avoiding premature convergence to local optima. This is done by adjusting the search step of every iteration by using the Coulomb force search strategy (CFCSS). The step vector of DA is modified so as to include the CFCSS as the step length instead of the linear step length. The position update formula then makes use of the modified step vector to update the position of the dragonflies. The velocity update equation of DA is replaced by a new equation, as shown in Table 2, where $a_{i}\left(t\right)$ represents the acceleration of all the dragonflies in the population.

In [24], a hybrid of DA and firefly algorithm called DA-FA is proposed. It improves the effectiveness of DA by enhancing its exploitation so as to prevent being trapped in local optima. It has an iterative level hybridization where DA is used as a global search and FA as a local search. Notably, the position update equation of the firefly algorithm is used for enhancing the exploitation.The position update equation of the firefly algorithm is employed, instead of updating the position of the dragonflies using the Levy flight mechanism when they have no neighbours. The new position update equation is shown in Table 2 where $\beta_{o}e^{-\gamma .r_{ij}^{2}}\left(X_{j}^{t}-X_{i}^{t}\right)$ represent the attraction of a firefly to a brighter firefly and $\propto (N_{rand}-0.5)$ is a random walk.

The hybrid DA and Nelder–Mead algorithm, called INMDA [26], increases the effectiveness of DA by improving its exploitation capability, so as to prevent convergence to local optima and to increase the solution accuracy. An iterative level hybridization is used where DA is first employed, followed by the improved Nelder–Mead algorithm, which further exploits the search region. A memory matrix is added to DA to record the best candidate solutions, and this matrix is then employed as an initial input vector of the improved Nelder–Mead algorithm. The improved Nelder–Mead algorithm is employed to further update the positions of the dragonflies after they have been updated by DA.

Some of the hybrids proposed successfully increase the effectiveness of DA by improving its exploitation, however, since increasing its efficiency is not their objective, the efficiency remains unchanged. Such hybrids of DA include OBLDA [13], hybrid DA-DE [20] and hybrid modified DA and whale optimization [30]. A detailed explanation of these algorithms is given next by considering information, which is not given in Table 2.

DA with opposition-based learning (OBL), called OBLDA, is proposed in [13]. It improves the performance of DA in terms of effectiveness by enhancing its exploitation phase, so as to better balance the exploration and exploitation phases of DA. After updating the position of dragonflies, OBL is applied to half of the population and it checks whether the position is better than its corresponding opposite and the fitter one is chosen as the position of the dragonfly. OBL is also used to improve the initialization of the population so as to obtain initial solutions with better fitness which helps in converging to the global optimum accurately. When the positions of a dragonfly is initialized in DA, the opposite of the position is found using OBL, and the fitter one is chosen as an individual of the initial population.

In [20], a hybrid DA-DE is proposed to increase the effectiveness of DA by improving its exploitation capability. DA is used as a global search since it has a good exploration capability, and differential evolution (DE) is incorporated as a local search to improve the exploitation and thus increase the accuracy of solutions. The average fitness of the population is first calculated in each iteration. Then, for each search agent, if the fitness is less than the average fitness, its position is updated using the step and position vectors of DA; otherwise, its position is updated using the mutation, crossover and selection operations of DE.

The hybrid modified DA and whale optimization [30] improves the effectiveness of the dragonfly algorithm by preventing the problem of being trapped in local optima. The algorithm has an iterative level hybridization where DA is first applied, and then the best solution obtained by DA is further updated using the whale optimization algorithm. There are two equations of the whale optimization algorithm which are added to DA as shown in Table 2, where ${\vec{D}}_{rand}$ represent random whales and $\vec{F}$, $\vec{E}$ and $\vec{C}$ represent coefficient vectors.

The hybrid DA-PSO algorithm [29] and IEDA [31] aim at increasing the effectiveness of DA by means of improving its exploitation, and they are successful in achieving their objective. However, the efficiency of the hybrid algorithms is lower than that of the original DA. We present an explanation on these algorithms by considering information which is omitted in Table 2.

The hybrid DA-PSO algorithm [29] solves the low exploitation problem of DA, so as to avoid getting trapped in local optima, thereby improving the effectiveness of the dragonfly algorithm. The algorithm has an iterative level hybridization where DA is first employed followed by PSO, so as to make use of the exploration of DA and exploitation of PSO. The best position obtained by DA is used as the global best position of PSO, which then further exploits the search space to obtain a better position. Hence, the velocity and position update equations of PSO are added to DA as shown in Table 2. The parameters $C_{1}$ and $C_{2}$ are acceleration coefficients, $rand_{1}$ and $rand_{2}$ are random values in range [0,1], *w* is the inertia weight, and $X_{DA}^{t+1}$ is the best position obtained by DA.

The hybrid DA-DE, called IEDA [31], improves the effectiveness of DA by enhancing its exploitation capability so as to surmount the problem of low accuracy of solutions. A division of labor strategy is employed so as to divide the population into two halves. One half is used for exploration, and the other one for exploitation. The global search capability of DA is used for exploration. The information exchange strategy of DE, together with an exemplar pool storing high quality individuals, are added and used for the exploitation population.

There is a hybrid algorithm on which we are working, and it also improves the effectiveness of DA by improving its exploitation phase. The algorithm [33] makes use of hill climbing algorithm as a local search. In each iteration, the position obtained by DA is further updated by hill climbing in order to obtain better positions. We did not include this algorithm in the tables and taxonomies in this paper because it is an ongoing work.

#### 3.4.2. Hybrids of DA Which Improve Its Effectiveness by Improving Exploration

Table 3 shows the hybrids of DA which have improved the effectiveness of the original DA by means of improving its exploration phase in terms of the algorithm used to improve DA, the way of improvement, the application that they have been used for, how much the effectiveness of DA is improved, and whether the efficiency is also improved or not. In the ’Improved effectiveness (%)’ column, the problem for which this improvement is achieved is shown within parentheses. In the ’way of improvement’ column, the phase and the equations or steps which are improved are shown.

Apart from increasing the effectiveness of DA by improving its exploitation phase, the effectiveness can also be increased by improving its exploration phase. The hybrid algorithms hybrid DADE [11], DA-ABC [25] and HLBDA [27] increase the effectiveness of DA by means of improving its exploration phase, and these algorithms also improve the efficiency of DA. Next, we present a detailed explanation on these algorithms by considering information which is not given in Table 3.

In [11], a hybrid DADE algorithm which is a hybrid of differential evolution and DA is proposed. The aim is to increase the effectiveness of DA by increasing the population diversity, and preventing the problem of getting trapped in local optima. A memory element is first incorporated in DA to store the best solutions obtained, namely the local best solution obtained by each dragonfly, and the global best solution obtained by the swarm. The mutation technique of differential evolution is then applied using the stored local and global best positions. The position update of DA is improved, since once the position has been updated by DA, the position is used as the target vector of differential evolution. Differential evolution then employs its mutation technique on the target vector by making use of the local and global best solutions to obtain a better position.

The hybrid of DA and artificial bee colony (ABC), called DA-ABC [25], improves the effectiveness of DA by enhancing its exploration capability. This is achieved by using DA as a global search, and in addition to that, the modified scout bee phase of ABC is also employed as a global search. The exploration is improved, since by employing two exploration phases, the search space and the diversity of the population are increased. The onlooker bee phase of ABC is then employed as a local search.

The hyper learning binary dragonfly algorithm (HLBDA) proposed in [27] improves the effectiveness of the binary dragonfly algorithm. A hyper learning strategy is employed to improve the exploration of BDA by taking into consideration the personal best and personal worst positions of each dragonfly and also the global best position of the population. Using the personal best and personal worst positions, the behaviors of finding food and fleeing enemies of the dragonflies are improved during the position update process. The equations to calculate the attraction to food and distraction from enemy are updated to include consideration of the personal best and personal worst positions as shown in Table 3. The parameters $Xpb_{i}$ and $Xpw_{i}$ are used to represent the personal best and personal worst positions, respectively. Furthermore, the personal and global best solutions improve the learning strategy. The position update formula is updated to consider the personal best and global best positions as shown in Table 3 where $r_{1}$ is a random number in the interval 0 and 1.

**Table 2:** Hybrids of DA which improve its effectiveness by improving exploitation.

Algorithm	Algorithm Used for Hybridisation	Way of Improvement		Application	Improved Effectiveness (%)	Efficiency
		Phase	Equation/Step			
Memory-based Hybrid DA (MHDA) [8]	PSO	Exploitation	**Equations to be improved:** (6), (7) **Added equations:** $\Delta X_{i}^{t+1}=w\Delta X_{i}^{t}+C_{1}r_{1}\left(DA-pbest_{i}^{t}-X_{i}^{t}\right)+C_{2}r_{2}\left(DA-gbest_{i}^{t}-X_{i}^{t}\right)$ $X_{i}^{t+1}=X_{i}^{t}+\Delta X_{i}^{t+1}$	CEC 2014 benchmark functions, Welded beam design problem	12.7% (welded beam design problem)	Improved
Coulomb force search strategy-based DA [12]	Coulomb force search strategy (CFCSS)	Exploitation	**Equation to be improved:** (6) **Modified equation:** $\Delta X_{i}^{t+1}=\left(sS_{i}+aA_{i}+cC_{i}+fF_{i}+eE_{i}\right)+w\Delta X_{i}^{t}+a_{i}\left(t\right)$	25 and a 72-bar space truss structure problem & Optimal design of the Bucket wheel reclaimer (BWR)	1.7% (72-bar space truss structure problem)	Improved
Hybrid DA and firefly (DA-FA) [24]	Firefly Algorithm (FA)	Exploitation	**Equation to be improved:** (8) **Modified equation:** $X_{i}^{t+1}=X_{i}^{t}+\beta_{o}e^{-\gamma .r_{ij}^{2}}\left(X_{j}^{t}-X_{i}^{t}\right)+\propto \left(N_{rand}-0.5\right)$	CEC 2019 benchmark functions, Wireless sensor networks localization problem	9.7% (CEC 10)	Improved
Hybrid DA and Nelder–Mead Algorithm (INMDA) [26]	Improved Nelder–Mead Algorithm	Exploitation	**Steps to be improved:** Lines 11 and 13 from Algorithm 1 **Step added (After line 15 in Algorithm 1):** Invoke INMDA	Training of multilayer perceptron	67.1% (function $F_{19}$)	Improved
DA with opposition-based learning (OBLDA) [13]	Opposition-based learning (OBL)	Exploitation	**Steps to be improved:** Lines 11 and 13 from Algorithm 1 **Step added (After lines 11 and 13 in Algorithm 1):** Select half of dragonflies from the population and apply OBL	Multilevel thresholding colour image segmentation	0.93%	No change
Hybrid DA-DE [20]	Differential evolution	Exploitation	**Steps to be improved:** Lines 11 and 13 from Algorithm 1 **Steps added (After lines 11 and 13 in Algorithm 1):** Evaluate fitness of dragonfly Compute average fitness of population If fitness of dragonfly is greater than average, apply mutation, crossover and selection operations of DE	Image segmentation	0.042% (penguin image using Otsu method, level 10)	No change
Hybrid modified DA and whale optimization [30]	Whale optimization algorithm	Exploitation	**Equations to be improved:** (6), (7) **Added equations:** $F=|\vec{E}.{\vec{D}}_{rand}-\vec{D}|$ $D\left(t+1\right)={\vec{D}}_{rand}-\vec{C}.\vec{F}$	Optimal scheduling of microgrid with islanding constraints	50.9% (test scenario 1)	No change
Hybrid DA-PSO [29]	PSO	Exploitation	**Equations to be improved:** (6), (7) **Added equations:** $\Delta X_{i}^{t+1}=w\Delta X_{i}^{t}+C_{1}.rand_{1}\left(pbest_{i}^{t}-X_{i}^{t}\right)+C_{2}.rand_{2}\left(X_{DA}^{t+1}-X_{i}^{t}\right)$ $X_{i}^{t+1}=X_{i}^{t}+\Delta X_{i}^{t+1}$	Multiobjective Optimal Power Flow (MO-OPF) problem	7.2E-4% (IEEE 30-bus system when considering only the fuel cost)	Lower efficiency
Hybrid DA-DE (IEDA) [31]	Differential Evolution	Exploitation	**Steps to be improved:** Lines 11 and 13 from Algorithm 1 **Steps added (After line 7 in Algorithm 1):** Select half of the population and apply information exchange strategy of DE	Optimal design of hybrid power active filter	36.6% (experiment case 1)	Lower efficiency

**Table 3:** Hybrids of DA which improve its effectiveness by improving exploration.

Algorithm	Algorithm Used for Hybridisation	Way of Improvement		Application	Improved Effectiveness (%)	Efficiency
		Phase	Equation/Step			
hybrid DADE [11]	Differential Evolution	Exploration	**Steps to be improved:** Lines 11 and 13 from Algorithm 1 **Steps added:** **After line 4 in Algorithm 1 -** Update personal and global best positions **After lines 11 and 13 in Algorithm 1 -** Apply mutation and crossover technique of DE	Benchmark mathematical functions & Welded beam design problem	2.1% (welded beam design problem)	Improved
Hybrid DA and ABC (DA-ABC) [25]	Artificial Bee Colony (ABC)	Exploration	**Steps to be improved:** Lines 11 and 13 from Algorithm 1 **Steps added (After line 14 in Algorithm 1):** Apply onlooker bee phase and modified scout bee phase of ABC	Training of multilayer perceptron	5.2% (’iris’ dataset)	Improved
Hyper Learning Binary Dragonfly Algorithm (HLBDA) [27]	Hyper Learning Strategy	Exploration	**Equations to be improved:**(4), (5), (7) **Modified equations:** $F_{i}=\left(\left(Xpb_{i}-X_{i}\right)+\left(X^{+}-X_{i}\right)\right)/2$ $E_{i}=\left(\left(Xpw_{i}+X_{i}\right)+\left(X^{-}+X_{i}\right)\right)/2$ $X_{i}^{t+1}\;=\{\begin{array}{cc} X_{i} & 0\leq r_{1}\leq pl\\ Xpb_{i}^{t} & pl\leq r_{1}\leq gl\\ Xf^{t} & gl\leq r_{1}\leq 1 \end{array}$	Feature selection	1.5% (’primary tumour’ dataset)	Improved
Hybrid DA-Opposition-based learning [23]	Opposition-based Learning (OBL)	Exploration	**Steps to be improved:** Lines 11 and 13 from Algorithm 1 **Step added (After line 14 in Algorithm 1):** Apply OBL to new position and select the best position	Real parameter function optimization	93.4% (function $f_{1}$)	No change
Hybrid DA-Simulated Annealing [22]	Simulated Annealing Algorithm	Exploration	**Steps to be improved:** Lines 11 and 13 from Algorithm 1 **Steps added (After line 14 in Algorithm 1):** Calculate retention probability using idea of simulated annealing If new position is better than original position, retain new position directly. Otherwise, retain new position based on retention probability	Flexible flow-shop scheduling	0.29% (’J10c10c3’ test data)	Lower efficiency
modified DA algorithm using Brownian motion [10]	Brownian motion	Exploration	**Equation to be improved:** (8) **Modified equation:** $X^{t+1}=X^{t}+h*rand\left(\right)*P_{g}$	Benchmark functions & Welded beam design problem	20% (welded beam design problem)	*Not considered*
BBO and Mexican hat wavelet DA (BMDA) [14]	BBO with Mexican hat wavelet	Exploration	**Equation to be improved:** (7) **Added equation:** $X_{i}=X_{i}+\propto \left(X_{j}-X_{i}\right)$	CEC2017 benchmark functions	65.0% (function $f_{i}$)	*Not considered*
Improved DA (IDA) [19]	Differential Evolution (DE)	Exploration	**Equations to be improved:** (7), (8) **Modified equations:** $X_{i}^{t+1}=c_{i}^{t}X_{i}^{t}+\Delta X_{i}^{t+1}$ $X_{i}^{t+1}=c_{i}^{t}X_{i}^{t}+Levy\left(d\right)\times X_{i}^{t}$ **Steps to be improved:** Lines 11 and 13 from Algorithm 1 **Steps added (After line 14 in Algorithm 1):** Use strategy of differential evolution on new position obtained by DA	Optimise the parameters of SVM	26.4%, 25.27%, 23.44% (3 types of prediction errors)	*Not considered*

The hybrid DA-opposition-based learning [23] and hybrid DA-simulated annealing algorithms [22] successfully increase the effectiveness of DA by means of improving its exploration. However, they are not able to increase the efficiency of DA. The hybrid DA-opposition-based learning [23] has the same efficiency as the original DA, while hybrid DA-simulated annealing algorithm [22] has lower efficiency. An explanation of these algorithms which is not given in Table 3 is presented next.

The hybrid DA-opposition-based learning algorithm [23] improves the effectiveness of DA by increasing the exploration of the search region. Opposition-based learning is first used during initialization to generate better initial positions for the artificial dragonflies. After the positions of the dragonflies are initialized, the opposite positions are found, and the fitter set of positions is selected as the initial positions for the population. In each iteration after the positions of the dragonflies have been updated by DA, the opposition-based learning technique is again employed on the new set of dragonflies generated to determine whether their opposite dragonflies have better positions. The ones with the better positions are then retained in the population.

The hybrid DA-simulated annealing algorithm in [22] enhances the effectiveness of DA by improving its ability to escape the local optimum. It makes use of the selection probability of simulated annealing to retain new individuals. If the latter is better than the one in the current iteration, it is retained directly; otherwise, it is retained with a certain probability. This is carried out after the position of a dragonfly has been updated by DA and unlike in DA, if the new position obtained is not better than the previous one, it is only retained with a certain probability.

The modified DA algorithm using Brownian motion [10], BMDA [14], and IDA [19] successfully achieve their objective of increasing the effectiveness of DA by improving its exploration phase. However, the efficiency of these algorithms is not considered or compared with the original DA, as their objective is only to improve the effectiveness of DA. Next, an explanation on these algorithms is given, by considering information which is not given in Table 3.

The modified DA algorithm using Brownian motion is proposed in [10] to increase the effectiveness of DA. Brownian motion is used instead of the Levy flight mechanism to update the position of dragonflies which have no neighborhood. This is in order to ameliorate the randomization stage of DA by preventing the Levy flight mechanism from overflowing the search area and interrupting random flights which are caused by its large searching steps, thereby improving the effectiveness of DA. The equation used to update the position of dragonflies instead of the Levy flight mechanism is shown in Table 3, where *h* and $P_{g}$ are calculated based on the motion time period of a dragonfly and its number of sudden motions in a specific time period.

The biogeography-based and Mexican hat wavelet DA (BMDA) proposed in [14] improves the effectiveness of DA by improving its exploration capability so as to prevent premature convergence to local optima. A new operator is first obtained by combining the migration process of the biogeography-based optimization (BBO) with Mexican hat wavelet in the mutation phase. It is then used to further update the solution obtained by DA after updating the position in each iteration. The operator makes use of the the migration of BBO to improve the quality of the solution and the mutation of BBO combined with Mexican hat wavelet to make the algorithm more stochastic. Hence, its exploration is increased, and it is able to prevent local optima. The new added equation is shown in Table 3, where the parameter $X_{j}$ represents the source of migration and ∝ is a random number in the interval [0,1].

In [19], an improved DA called IDA is proposed to enhance the effectiveness of DA. It prevents the low convergence accuracy caused by the random walk strategy when the dragonflies do not have a neighbourhood by using an adaptive learning factor. The adaptive learning factor is introduced in the position vectors of DA; the one used when dragonflies have neighbours and the one used when they have no neighbours as shown in Table 3, where the parameter $C_{i}^{t}$ represents the adaptive learning factor. Moreover, the strategy of differential evolution is used to diversify the population, thereby preventing the problem of being stuck in local optima. This strategy is employed in each iteration after the position is updated by the modified position vector of DA.

#### 3.4.3. Hybrids of DA Which Improve Its Effectiveness by Improving Both Exploitation and Exploration

Table 4 shows the hybrids of DA which have improved the effectiveness of the original DA by means of improving both its exploitation and its exploration phases in terms of the algorithm used to improve DA, the way of improvement, the application that they have been used for, how much the effectiveness of DA is improved, and whether the efficiency is also improved or not. In the ’Improved effectiveness (%)’ column, the problem for which this improvement is achieved is shown within parentheses. In the ’way of improvement’ column, the phase and the equations or steps which are improved are shown.

Some of the hybrids of DA which have been proposed increase the effectiveness of DA by improving both its exploitation and exploration phases. Such hybrids include the QGDA [16] and EOEDA [21], and these two hybrids of DA also increase its efficiency. An explanation of these algorithms which is not presented in Table 4 is given next.

The quantum-behaved and Gaussian mutational DA (QGDA) proposed in [16] aims at improving the effectiveness of DA. It better balances the exploration and exploitation of DA by expanding the state space and increasing the diversity. The Gaussian mutation mechanism is used to augment the population diversity and quantum rotation gate is employed to increase the search space. After the position of a dragonfly is updated by DA, the guassian mutation and quantum rotation gate are employed to obtain a better position.

The improved DA based on elite opposition learning and exponential function steps, EOEDA [21], improves the effectiveness and efficiency of DA. It enhances both the exploration and exploitation of DA so as to prevent the problem of local optimum and to increase the accuracy and convergence rate. The elite opposition-based learning is incorporated to diversify the population by expanding the search scope. In each iteration, the elite opposition based solution of each dragonfly is found and then the best solutions are chosen as the population. Moreover, an exponential function step is used to replace the step length in the step vector, which enhances both the local and global search capability and accelerates the convergence rate. The position of the dragonflies is then updated using the modified step vector, which makes use of the exponential function step.

The EOBL [17] algorithm, hybrid improved DA [28] and DA-GA [32], improve both the exploitation and exploration phases of DA, thereby increasing its effectiveness. However, the efficiency of these algorithms is lower than that of the original DA. Next, we present an explanation of these algorithms which is not given in Table 4.

The hybrid DA-DE algorithm with chaotic maps and elite opposition-based learning (EOBL) [17] improves the effectiveness of DA by better balancing the exploration and exploitation phases, and by enhancing the initialization of the population. Chaotic maps and EOBL are first used to provide fitter initial positions for the dragonflies by increasing the randomness of the population. In the updating phase, differential evolution is employed as a local search technique to increase the exploitation of DA, thereby improving the accuracy of solutions. The average fitness of the population is first calculated in each iteration. Then, for each search agent, if the fitness is less than the average fitness, its position is updated using the step and position vectors of DA; otherwise, its position is updated using the mutation, crossover and selection operations of DE.

The hybrid improved DA [28] improves the effectiveness of DA. It aims at balancing the local and global search capabilities and at enhancing the exploitation of DA. This is achieved by using dynamic weights; namely, the separation, alignment, cohesion and enemy weights, which decrease with iterations so as to strengthen the exploration in the earlier iterations and exploitation during later iterations, thereby balancing the exploration and exploitation capabilities. Moreover, the exploitation is further enhanced by applying the approach of quantum optimal solution on the current and optimal solutions. The improved velocity update formula is given in Table 4, where $C_{1}$ and $C_{2}$ are the cognitive and social parameters, $r_{1}$ is a random number in the interval [0,1], $P_{i}^{t}$ is the best fitness of the dragonfly and $P_{G}$ is the best fitness of the population.

The hybrid of DA and genetic algorithm (GA), called DA-GA [32] is proposed to improve the effectiveness of DA. It balances the global and local searching capabilities of DA, so as to prevent the local optima problem. The population is divided into two halves. In the first half of the population, the dragonflies update their positions using DA, and in the other half, the position of the dragonflies is updated using GA. The new population then consists of all the dragonflies of which the positions have been updated using DA and those of which the positions have been updated using GA.

#### 3.4.4. Hybrids of DA Which Improve Its Effectiveness by Improving Initialization

Table 5 shows the hybrids of DA which have improved the effectiveness of the original DA by means of improving the initialization phase in terms of the algorithm used to improve DA, the way of improvement, the application that they have been used for, how much the effectiveness of DA is improved, and whether the efficiency is also improved or not. In the ’Improved effectiveness (%)’ column, the problem for which this improvement is achieved is shown within parentheses. In the ’way of improvement’ column, the phase and the equations or steps which are improved are shown.

Another way by which the effectiveness of the original DA is improved is by improving its initialization stage. The chaotic DA [9] has employed this method to increase the effectiveness of DA, and it successfully increases its efficiency as well. Next, we present an explanation on these algorithms by considering information which is not given in Table 5.

The chaotic DA is proposed in [9] to increase the effectiveness and efficiency of DA by accelerating the convergence rate and avoiding local optima. Chaotic maps are utilized to generate the weights for the position update parameters instead of using random initial values. The weights for the factors used in the step vector, namely separation, alignment, cohesion, food factor and enemy factor, are then based on chaotic values. This allows the algorithm to better update the step vector. Hence, the chaotic maps are used for better adjustment of the dragonflies’ movement through the search space. The modified step vector is given in Table 5, where B(i) is the value of a chaotic map at the *i-th* iteration.

The SC-DA algorithm [15] and the hybrid DA-modified conjugate gradient method [18] improve the effectiveness of the original DA by improving its initialization stage. However, since improving the effectiveness is their only objective, their efficiency is not considered or compared to the efficiency of the original DA. An explanation of these algorithms which is not given in Table 5 is presented next.

The chaotic DA based on sine-cosine mechanism (SC-DA) [15] is proposed to improve the effectiveness of DA by improving its accuracy. The singer chaos theory is first used for better initializing the position of the population, and then the sine-cosine mechanism is used to update the position of the artificial dragonflies in every iteration. The position update formula is given in Table 5, where $d_{2}$ is a random number between 0 and 2π, $d_{3}$ and $d_{4}$ are random numbers in the interval [0,1], *d* is based on the number of iterations, *d* is the threshold which is set at 0.5, and $W_{i}^{t}$ is the position of the best solution.

**Table 4:** Hybrids of DA which improves its effectiveness by improving both exploitation and exploration.

Algorithm	Algorithm Used for Hybridisation	Way of Improvement		Application	Improved Effectiveness (%)	Efficiency
		Phase	Equation/Step			
Quantum behaved and Gaussian mutational DA (QGDA) [16]	Gaussian Mutation Mechanism and Quantum Rotation Gate	Exploration and Exploitation	**Steps to be improved:** Lines 11 and 13 from Algorithm 1 **Steps added (After line 14 in Algorithm 1):** Update position of dragonfly using Gaussian mutation Perform quantum gate operation	CEC 2014 benchmark functions	99.9% (function $f_{1}$)	Improved
Improved DA based on elite opposition learning and exponential function steps (EOEDA) [21]	Elite Opposition Learning and Exponential Function Steps	Exploration and Exploitation	**Equation to be improved:** (7) **Modified equation:** $X_{i}^{t+1}=X_{i}^{t}+\left(rand-0.5\right).2^{rand}.\Delta X_{i}^{t+1}$ **Step to be improved:** Line 11 from Algorithm 1 **Step added (After line 14 in Algorithm 1):** Generate elite opposition solution of new solution Compare new solution and elite opposition solution and select the best one	Numerical optimization of standard functions	100% (function $f_{1}$)	Improved
Hybrid DA-DE algorithm with chaotic maps and elite opposition-based learning [17]	DE, Chaotic Maps and Elite-Opposition based Learning	Exploration and Exploitation	**Steps to be modified:** Line 11 and 13 from Algorithm 1 **Steps added (After lines 11 and 13 in Algorithm 1):** Evaluate fitness of dragonfly Compute average fitness of population If fitness of dragonfly is greater than average, apply mutation, crossover and selection operations of DE	Multilevel thresholding image segmentation	0.037% (’bridge’ image using Otsu’s method)	Lower efficiency
Hybrid improved DA [28]	Approach of Quantum optimal solution and Use of Dynamic weights	Exploration and Exploitation	**Step to be improved:** Line 6 from Algorithm 1 **Modified step:** Update weights using $f_{t}=Init\left(1-\frac{1}{1+e^{-0.1(t-50)}}\right)$ **Equation to be improved:** (6) **Modified equation:** $\Delta X_{i}^{t+1}=\left(sS_{i}+aA_{i}+cC_{i}+fF_{i}+eE_{i}\right)+$ $w\Delta X_{i}^{t}+C_{1}r_{1}\left(MP_{i}^{t}\right)*ln\left(1/u\right)$ $+C_{2}\left(1-r_{1}\right)\left(P_{G}-X_{i}^{t}\right)$	Feature selection	2.5% (’Arrhythmia’ dataset)	Lower efficiency
Hybrid DA and GA (DA-GA) [32]	Genetic Algorithm (GA)	Exploration and Exploitation	**Step to be improved:** Line 11 and 13 from Algorithm 1 **Added and modified steps (After line 4 in Algorithm 1):** Divide population in half Update position of first half using DA Update position of second half using GA Form new population by taking both halves	Optimal power flow problem	0.084% (line outage between buses 6 and 26 in the 38 bus RDS)	Lower efficiency

**Table 5:** Hybrids of DA which improve its effectiveness by improving initialization.

Algorithm	Algorithm Used for Hybridisation	Way of Improvement		Application	Improved Effectiveness (%)	Efficiency
		Phase	Equation/Step			
Chaotic DA [9]	Chaos theory	Initialization	**Step to be improved:** Line 6 in Algorithm 1 **Step added (before line 6 in Algorithm 1):** Calculate the value of chaotic map **Equation to be improved:** (6) **Modified equation:** $\Delta X_{i}^{t+1}=(B\left(i\right)S_{i}+B\left(i\right)A_{i}+B\left(i\right)C_{i}+$ $B\left(i\right)F_{i}+B\left(i\right)E_{i})+B\left(i\right)\Delta X_{i}^{t}$	Feature selection	18.1% (’Irritant effect’ dataset)	Improved
DA with opposition-based learning (OBL) [13]	Opposition-based learning (OBL)	Initialization	**Step to be improved:** Line 1 in Algorithm 1 **Step added (After line 1 in Algorithm 1):** Compute the opposite of each solution using OBL Select the fitter solution to form the initial population	Multilevel thresholding colour image segmentation	0.93%	No change
Hybrid DA-Opposition-based learning [23]	Opposition-based learning (OBL)	Initialization	**Step to be improved:** Line 1 in Algorithm 1 **Step added (After line 1 in Algorithm 1):** Compute the opposite of each solution using OBL Select the fitter solution to form the initial population	Real parameter function optimization	93.4% (function $f_{1}$)	No change
Hybrid DA-DE algorithm with chaotic maps and elite opposition-based learning [17]	DE, Chaotic Maps and Elite-Opposition based Learning	Initialization	**Step to be improved:** Line 1 in Algorithm 1 **Modified step:** Generate initial population using chaotic maps Generate elite opposition population Select the best positions as the initial population	Multilevel thresholding image segmentation	0.037% (’bridge’ image using Otsu’s method)	Lower efficiency
Chaotic DA based on sine-cosine mechanism (SC-DA) [15]	Chaos theory and Sine-Cosine Mechanism	Initialization	**Step to be improved:** Line 1 in Algorithm 1 **Modified step:** Generate initial population using singer chaos **Equation to be improved:** (7) **Modified equation:** $X_{i}^{t+1}\;=\{\begin{array}{cc} X_{i}^{t}+d_{1}sin\left(d_{2}\right)\left|d_{3}W_{i}^{t}-X_{i}^{t}\right|, & d_{4}<d\\ X_{i}^{t}+d_{1}cos\left(d_{2}\right)\left|d_{3}W_{i}^{t}-X_{i}^{t}\right|, & d_{4}\geq d \end{array}$	Numerical benchmark functions	83.3% (’Sphere’ function)	*Not considered*
Hybrid DA-Modified Conjugate Gradient [18]	Modified Conjugate Gradient Method	Initialization	**Step to be improved:** Line 1 in Algorithm 1 **Modified step:** Generate initial population using Modified Conjugate Gradient	Standard numerical functions	29.2% (function $F_{5}$)	*Not considered*

The hybrid DA-modified conjugate gradient method [18] improves the effectiveness of DA by generating a better initial population. The modified conjugate gradient method is employed to generate the initial positions for the population of artificial dragonflies, and then the dragonfly algorithm is applied for updating their positions.

The DA with opposition-based learning (OBLDA) [13], hybrid DA-DE algorithm with chaotic maps and elite opposition-based learning [17] and hybrid DA-opposition-based learning [23], which have been discussed earlier, also improve the effectiveness of the original DA by means of improving its initialization stage. However, these hybrids of DA do not increase its efficiency. The efficiency of OBLDA [13] and hybrid DA-DE algorithm with chaotic maps and elite opposition-based learning [17] are the same as the original DA, while that of the hybrid DA-opposition-based learning [23] is lower than the original DA.

## 4. Applications of DA and Hybrids

Both the original DA, BDA and MODA and their hybrids have been applied to numerous applications in a variety of areas. These applications are presented in this section based on their respective domains.

Table 6 shows the applications of DA and its hybrids in different domains.

Based on Table 6, it can be seen that the dragonfly algorithm is useful in various domains. It has been applied predominantly in the field of machine learning, electrical engineering, optimal design, numerical optimization, and digital image processing. It also has numerous applications in networking, resource allocation, and mechanical engineering.

### 4.1. Optimal Design

The Dragonfly Algorithm and its hybrids have been used for optimal designs, by either optimizing an objective function or optimizing certain parameters. In [34], DA is used to determine the optimal set of array parameters for the concentric circular antenna array (CCAA), so as to minimize the maximum sidelobe level (MSL) of the radiation pattern. In [35], DA is used in the optimal design of infinite impulse response (IIR) to get the optimal set of filter coefficients by minimizing the cost of an objective function and in [36], it is used in the orthotropic infinite plates optimization for optimization of the parameters of the stress analysis of perforated orthotropic plates.

The hybrids of DA have also been used for optimal designs. The MHDA algorithm [8], the modified DA using Brownian motion [10] and hybrid DADE [11] have been used for the welded beam design problem, which consists of minimizing the fabrication cost by obtaining an optimal set of structural parameters of the beam. Moreover, the Coulomb force search strategy-based DA [12] has been applied to the bucket wheel reclaimer (BWR) optimization to determine the optimal parameters to optimize the structure of a BWR and to a 25 and a 72-bar space truss structure problem, Chaotic DA based on sine-cosine mechanism (SC-DA) [15] has been used for the cantilever beam design problem to optimize the parameters of the beam, such that its weight is minimized and Hybrid DA-DE (IEDA) [31] has been used for the optimal design of a hybrid power active filter. The quantum-behaved and Gaussian mutational DA (QGDA) [16] has been used for the the welded beam design problem, the multiple disk clutch brake design problem and I-beam design problem.

### 4.2. Electrical Engineering

In the field of electrical engineering, DA and its hybrids have been used for various problems, most of which are related to power systems. In [37], DA has been applied to the economic load dispatch problem in power systems, which consists of minimizing the generation cost while satisfying constraints such as ramp rate, demand and generator operating limit; in [38], it has been applied to the static economic dispatch problem incorporating solar energy; in [39], it has been applied to the combined economic emission dispatch problem, and in [40], it has been applied to the dynamic economic dispatch problem. In [41,42], DA has been applied to the automatic generation control problem to optimize the gains of the controller. Furthermore, DA has been used in power transmission systems [43] for optimizing the size and cost of static var compensator, in PV panel [44] to maximize the tracking of solar power, in photovoltaic system [45] to track the global maximum power point (GMPP) and in Photovoltaic-biomass system [46] to find the optimal size and cost of grid-integrated renewable energy resources. DA has also been applied to the optimal reactive power dispatch problem for minimization of the power loss in transmission lines in [47] and to the load frequency control of electric power generating system to tune the gains and fractional order parameters of the controller in [48].

The multi-objective version of DA, MODA has been applied to wind-solar-hydro power scheduling in [49] to provide an optimal scheduling model and to multi-objective optimal power flow problem in [50] for minimizing total fuel cost, real power loss, total emission, and voltage deviation.

The hybrid DA-PSO [29] has been used for the multiobjective optimal power flow problem to optimize selected objective functions, while satisfying a set of equality and inequality constraints and the hybrid DA-GA [32] has been applied to optimal power flow problem to compute the locational marginal prices (LMP) for improved reliability.

**Figure 7:**
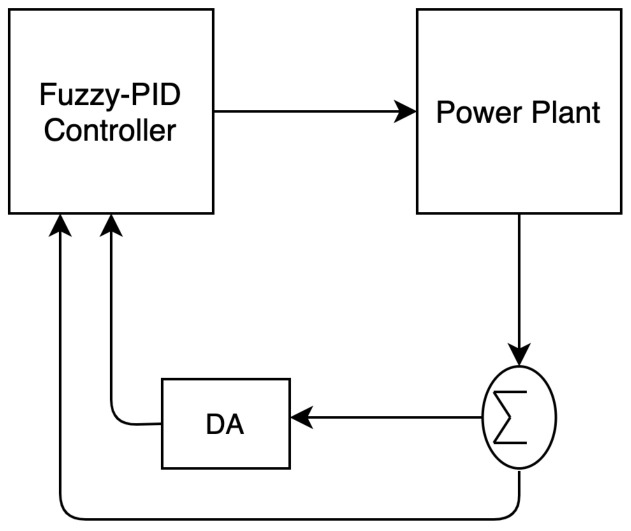
Optimization of fuzzy PID controller using DA [41].

Figure 7 shows how the dragonfly algorithm is used for solving the automatic generation control problem in [41] by optimizing the parameters of the fuzzy PID controller.

### 4.3. Networking

In the field of networking, DA has been employed to choose the most optimal cluster heads in a radio frequency identification (RFID) network in [51,52]. In [53], it has been used for the range-based wireless node localization to obtain the location of nodes in a network which are randomly deployed over a designated area. DA has also been applied in the internet of vehicles to optimize the cluster-based packet route in [54].

The binary version of DA, BDA, has been applied to an optimal network power expansion planning to minimize the costs involved in the development of the network in [55].

The hybrid DA-FA algorithm [24] has been used for the wireless sensor networks localisation problem to locate unknown nodes using known position of anchor node.

**Figure 8:**
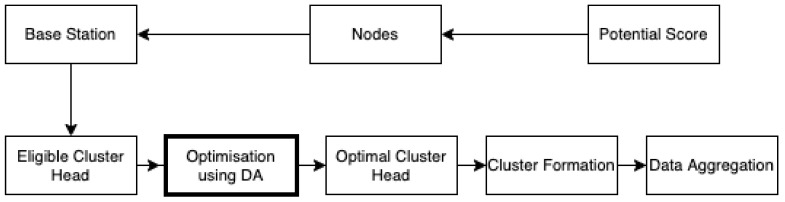
Cluster head selection in RFID network using DA [52].

Figure 8 depicts how the dragonfly algorithm is applied in RFID networks for the optimal cluster head selection and formation by using the separation, alignment, and cohesion techniques of DA.

### 4.4. Mechanical Engineering

In the field of mechanical engineering, the multi-objective version of DA, MODA, has been applied in the grinding process to maximize the final surface quality and minimize the cost and total process time in [56].

### 4.5. Machine Learning

DA and its hybrids have been used for various machine learning applications. DA has been used for the training of a multi-layer perceptron (MLP) in [57,58], which are then used for analysing the bearing capacity of a two-layered soil and for the classification of sonar target with high accuracy, respectively. It has also been applied for optimization of the parameters of support vector machines (SVM) in [59,60,61,62], which are then used for classification, regression, prediction and prediction, respectively. Moreover, DA has been used for the training of artificial neural networks (ANN). In [63], it is used to speed up the training process of an ANN used for MRI brain image classification. In [64], DA is used for minimizing the mean square error (MSE) during the training of an ANN, and in [65], it is used to determine the optimal parameters of a long and short-term memory neural network. It has also been applied to an extreme learning machine (ELM) prediction to optimize the weights and biases, and to minimize the number of nodes in the hidden layer in [66].

The binary version of DA, BDA, has been used in feature selection to obtain an optimal subset of features in [67,68].

In [9], the chaotic DA has been used in feature selection for minimizing the size of the selected features, so as to maximize classification performance. Hyper learning BDA [27], quantum-behaved and Gaussian mutational DA (QGDA) [16] and the hybrid improved DA [28] have also been used in feature selection to obtain an optimal subset of features. The hybrid DA-ABC [25] and hybrid Nelder–Mead algorithm and DA [26] have been used for the training of a multilayer perceptron to obtain the most optimal weights and biases for the MLP. Furthermore, improved DA (IDA) [19] has been applied to optimise the parameters for a support vector machine which was used for prediction.

**Figure 9:**
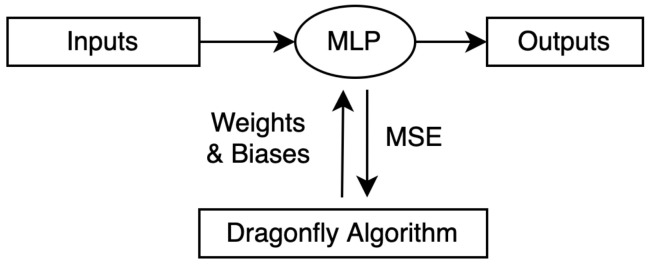
Training of MLP using DA [58].

Figure 9 shows how the dragonfly algorithm is used for the training of an MLP in [58], which is then used for the classification of solar targets. DA optimizes the weights and biases of the MLP by using the mean square error (MSE) as the objective function.

### 4.6. Resource Allocation

DA and its hybrids have also been applied for optimal resource allocation. In [69], DA is used for the optimal resource allocation in cloud computing by optimizing parameters like load balance, execution time and response time for optimal allocation of resources to tasks and to establish load balance. In [70], it has been used to optimally allocate generators and capacitors in a distribution system, and in [71], it has been used for the distributed generation (DG) placement in distributed networks to obtain the optimal DG units size and placement.

The hybrid modified DA and whale optimization [30] were applied to the optimal scheduling of microgrid with islanding constraints. In [22], the hybrid DA-simulated annealing is applied to the flexible flow-shop scheduling to optimize the online sequence for better scheduling.

### 4.7. Digital Image Processing

DA and its hybrids have also been useful in the domain of digital image processing. DA has been applied to medical image watermarking [72] and to medical image registration [73] to determine the most effective pixel that follows an objective function, and to determine the optimal transformation parameters respectively. It has also been used in 3D magnetic resonance imaging as a search method in the level set technique in [74]. In [75], DA is applied to multi-level thresholding for image segmentation to find the optimal thresholds.

The binary version of DA, BDA, has been applied to color visual cryptography in [76] to find the optimal color levels for the encryption process.

The hybrids of DA, namely DA with opposition-based learning [13], hybrid DA-DE with chaotic maps and elite opposition-based [17] and hybrid DA-DE [20] have been used for multilevel thresholding image segmentation to determine the optimal threshold values.

### 4.8. Numerical Optimization

Some of the hybrids of DA have only been used for numerical optimization, usually by using some benchmark functions such as the CEC 2017 benchmark functions to determine the optimal values for the functions. These hybrid algorithms include BMDA (biogeography-based and Mexican hat wavelet DA) [14], Hybrid DA-modified conjugate gradient method [18], improved DA based on elite opposition learning and exponential function steps [21], and hybrid DA-opposition-based learning [23].

The MHDA algorithm [8], modified DA algorithm using Brownian motion [10], hybrid DADE [11], chaotic DA based on sine-cosine mechanism (SC-DA) [15] and quantum-behaved and Gaussian mutational DA (QGDA) [16] have been used for numerical optimization, in addition to other applications.

### 4.9. Other Applications

Apart from the applications mentioned in the previous sections, DA has been applied to the vehicle routing problem with time window constraints (VRPTW) in [77] to find an optimized route and to the open loop nonlinear dynamic systems control in [78] to find the optimal parameters.

BDA has been applied to solve the 0–1 knapsack problem in [79].

## 5. Discussion, Challenges and Future Directions

The dragonfly algorithm and its hybrids are useful in various domains, and they have a multitude of applications. In several applications, it has been noted that DA and its hybrids have a higher performance in comparison to other swarm intelligence algorithms. However, they also have some limitations.

In Section 5.1, we discuss the performance of DA in terms of effectiveness, as compared to other swarm intelligence algorithms in multiple applications. In Section 5.2, we discuss its performance in terms of efficiency as compared to other swarm intelligence algorithms in various applications. In Section 5.3 and Section 5.4, the limitations of the dragonfly algorithm and some possible solutions to improve its performance in terms of effectiveness and efficiency respectively are provided. In Section 5.6, some of the limitations of the hybrids of DA that have been proposed to enhance the performance of DA are presented. In Section 5.7, the prospect for having different versions for the hybrids of DA is discussed.

### 5.1. Effectiveness of DA

The dragonfly algorithm has a higher effectiveness; that is, it provides better solutions than numerous swarm intelligence algorithms for several applications that it has been used for. For example, in [34], DA provides better solutions than symbiotic organisms search (SOS), evolutionary programming (EP), biogeography-based optimization (BBO), uniform array, sequential quadratic programming (SQP), firefly algorithm (FA), opposition-based gravitational search algorithm (OGSA), and cat swarm optimization (CSO), and in [38], it provides better solutions than genetic algorithm (GA), biogeography-based optimization (BBO), particle swarm optimization (PSO), and differential evolution (DE). In [68], the binary version of DA, that is, BDA, provides better results than GA, binary bat algorithm (BBA), binary PSO, binary gray wolf optimizer (BGWO) and binary gravitational search algorithm (BGSA), and in [79], it provides better results than harmony search, cohort intelligence algorithm, binary cuckoo search, quantum-inspired cuckoo search, and PSO. In [56], the multi-objective version of DA, that is MODA, provides better solutions than the non-dominated sorting genetic algorithm (NSGA-II), and in [49], it provides better solutions than NSGA-III.

To provide a comparison between the effectiveness of DA and some other swarm intelligence algorithms, the results provided by DA, PSO, grey wolf optimizer (GWO) and whale optimization algorithm (WOA) in solving some test functions are given in Table 7. The benchmark test functions, TF1 to TF13, are taken from [2]. The results provided by DA and PSO in solving the test functions are also taken from [2]. However, the results obtained by GWO and WOA are based on our own experiments. Table 7 shows the average cost of the objective function obtained by DA, PSO, GWO and WOA for test functions TF1 to TF13. The maximum number of iterations for all algorithms is kept at 500, and the number of search agents is kept at 30.

From Table 7, it can be seen that, for some of the test functions, DA provides better results than PSO, GWO, and WOA. For example, for test functions, TF1, TF2, TF3, TF4, TF5, and TF10, DA provides better results than PSO, for test functions TF6, TF8, and TF13, DA provides better results than GWO, and for test functions TF3, TF4, TF5, TF6, TF12 and TF13, DA provides better results than WOA. However, for some test functions, PSO, GWO, or WOA provide better results than DA.

### 5.2. Efficiency of DA

In terms of efficiency, DA has a higher efficiency; that is, it has a higher convergence rate in some applications as compared to other swarm intelligence algorithms. However, this is not the case for all the applications. For example, in [37], DA has a higher convergence rate in comparison to crow search algorithm, particle swarm optimization, biogeography-based optimization, ant lion optimizer, genetic algorithm and oppositional real-coded chemical reaction optimization. In [55], BDA has a lower execution time than GA and Tabu Search. In [49], MODA has a higher convergence rate than NSGA-III. However, in [35], DA has a lower convergence rate than particle swarm optimization (PSO), cat swarm optimization (CSO) and bat algorithm (BA), and in [73], it requires more computing time than artificial bee colony (ABC) and PSO.

### 5.3. Limitations of DA—Effectiveness

#### 5.3.1. Low Exploitation

DA has a high exploration which is beneficial for exploring the search space and finding promising regions. However, it has a low exploitation, which can lead to low accuracy of solutions. This limitation can be circumvented by increasing the exploitation of DA. Local search algorithms including direct search methods can be integrated as a local search in DA to increase its exploitation. Other swarm intelligence algorithms with a high exploitation can also be used as a local search to improve the exploitation of DA.

#### 5.3.2. Local Optima

Although DA has a good global search which is beneficial for avoiding local optima, it can still encounter the problem of falling in local optima, and hence the global optimum solution cannot be found. This possibility can be avoided by integrating techniques in the dragonfly algorithm, which will enable it to identify when the population is stuck in a local optimum and to allow the artificial dragonflies to get out of the local optimum.

#### 5.3.3. Low Accuracy of Solutions

DA can sometimes provide solutions with low accuracy. Although this can be avoided by overcoming the low exploitation of DA, other techniques can also be employed to increase its accuracy. For example, methods to generate better initial positions for the artificial dragonflies in the search space can be used. This will allow the population to have a better search, and consequently find solutions with a high accuracy.

### 5.4. Limitations of DA-Efficiency

In some applications such as in [35,73], it is found that DA has a lower efficiency as compared to other swarm intelligence algorithms; that is, it requires more time to converge to the global optimum solution.

The convergence rate of DA can be increased so as to increase the efficiency of the algorithm. Techniques to increase its convergence rate include methods to better update the velocity and position of the dragonflies in the search space and to accelerate the global and local search.

### 5.5. Way of Improvement of the Hybrids of DA

Most of the hybrids focus on improving the exploitation phase of DA, such as the MHDA [8], Coulomb force search strategy-based DA [12], DA-FA [24], hybrid DA and Nelder–Mead algorithm (INMDA) [26], and other hybrids, as shown in Figure 6 and Table 2. This is because DA has a quite good exploration phase, but its exploitation is limited. Hence, these hybrids make use of the exploration of DA and they enhance its exploitation. This is usually done by adding equations after the position has been updated by DA to further improve the position.

Some of the hybrids which have been proposed, such as hybrid DADE [11], DA-ABC [25], HLBDA [27] and others, as shown in Figure 6 and Table 3, improve the exploration phase of DA. This is to allow the algorithm to search through more different regions of the search space, thereby increasing the chance of finding the region of the global optimal solution. This is usually done by modifying the equations or adding some equations or steps while updating the position of the search agents.

Another way in which some the hybrids improve the performance of DA is by improving its initialization stage such as the chaotic DA [9], DA with opposition-based learning (OBL) [13], hybrid DA-opposition-based learning [23] and other algorithms shown in Figure 6 and Table 5. This is done so as to use some methods to generate the initial positions of the search agents instead of using random initial positions. By generating better initial positions, the positions of the search agents will be updated in a better way in the subsequent iterations.

### 5.6. Limitations of the Hybrids of DA

The hybrids of the dragonfly algorithm are all more effective than the original DA; that is, they provide better solutions than the original DA and some of the hybrids are more efficient than the original DA; that is, they have a higher convergence rate. However, some of the hybrids still have certain limitations.

The hybrid DA-DE with chaotic maps and elite opposition-based learning [17], the hybrid DA-simulated annealing [22], the hybrid DA-PSO [29], hybrid DA-DE [31] and hybrid DA-GA [32] all have a lower convergence rate than DA.

The hybrid improved DA [28] has a high computational complexity and lower convergence rate than BDA. The MHDA [8] has a high computational complexity.

Moreover, the chaotic DA [9] has a low stability, since a lot of parameters are used in the position update, along with their corresponding weights. The modified DA using Brownian motion [10] still has the possibility of getting stuck in local optima, and the communication between the dragonflies might be reduced, which will restrict discovery.

### 5.7. Nature of Problem

The dragonfly algorithm has three versions: one for continuous and single-objective problems, one for binary and single-objective problems, and one for continuous and multi-objective problems. However, we found that most of the hybrids that have been proposed cater for only one type of problem. Hence, the other versions of these hybrid algorithms can be proposed so as to allow them to be used for solving different types of problems, including continuous problems, binary or discrete problems, and multi-objective problems.

## 6. Conclusions and Future Work

The dragonfly algorithm, a recently proposed swarm intelligence algorithm, has been applied in numerous applications, and it is shown to have a higher performance as compared to other swarm intelligence algorithms.

There exists only a few surveys about DA, its applications and its hybrids and they are limited in certain aspects. For example, there is no analysis of the limitations of the proposed hybrids of DA and no categorization of the hybrids according to the type of problem that they have been applied to, their objective, or the method that they employ to achieve their objective.

In this paper, we present a survey on the dragonfly algorithm, particularly focusing on its applications and hybrids. A background on DA is first presented, followed by a review of its hybrids categorized by the type of problem that they have been applied to. The performance of the hybrids is then analyzed in terms of effectiveness and efficiency and the method that has been utilized. The applications of both the original DA and the hybrids are then presented and a discussion on their performance is given. Some challenges of the dragonfly algorithm and future directions are also given.

From the review of the dragonfly algorithm, it can be deduced that DA is useful in numerous applications, and it has a higher performance in comparison to other swarm intelligence algorithms. Nonetheless, it has some limitations which can be improved, and the existing hybrids also have certain limitations. Hence, new methods can be proposed to enhance both the effectiveness and the efficiency of the dragonfly algorithm.

For future work, the performance of the hybrids of DA can be compared to that of the hybrids of other swarm intelligence algorithms in general. Moreover, hybrids of other swarm intelligence algorithms, which have employed similar techniques as the hybrids of DA to improve the original algorithms, can be compared. Hybrids of the binary DA can be compared to the hybrids of other binary or discrete algorithms. Similarly, hybrids of the multi-objective version of DA can be compared to the hybrids of the multi-objective version of other algorithms. Furthermore, all the hybrids can be applied on a common benchmark problem, so that their performance in solving the same benchmark problem can be compared.

## Figures and Tables

**Table 1:** Comparison between our survey and previous surveys.

	Our Survey	[3]	[4]	[5]	[6]
Background on DA	✓	✓	✓	✓	✓
Applications of DA based on domain	✓	✓	✓	✓	✓
Analysis of the performance of DA as compared to other swarm intelligence algorithms	✓		✓	✓	✓
Consideration of the limitations of DA and proposed future directions	✓	✓	✓	✓	✓
Analysis of the performance of the hybrids of DA as compared to original DA	✓	✓	✓	✓	✓
Consideration of the methods employed to enhance the original DA obtaining the hybrids	✓	✓	✓	✓	✓
Analysis of the limitations of the hybrids of DA	✓				
Categorization of hybrids according to the type of problem	✓				
Taxonomies to categorize the hybrids of DA according to the performance improvement (effectiveness, efficiency)	✓				
Taxonomies of hybrids of DA according to effectiveness improving method	✓				

**Table 6:** Applications of DA and its hybrids in different domains.

Domain	Applications
Optimal Design	[8,10,11,12,15,16,31,34,35,36]
Electrical Engineering	[29,32,37,38,39,40,41,42,43,44,45,46,47,48,49,50]
Networking	[24,51,52,53,54,55]
Mechanical Engineering	[56]
Machine Learning	[9,16,19,25,26,27,28,57,58,59,60,61,62,63,64,65,66,67,68]
Resource Allocation	[22,30,69,70,71]
Digital Image Processing	[13,17,20,72,73,74,75,76]
Numerical Optimization	[8,10,11,14,15,16,18,21,23]
Other Applications	[77,78,79]

**Table 7:** Results provided by the algorithms for test functions.

Test Function	Average Cost of Objective Function			
	DA	PSO	GWO	WOA
TF1	2.85E-18	4.2E-18	6.1914E-57	3.9083E-75
TF2	1.49E-05	0.003154	3.126E-33	1.6851E-52
TF3	1.29E-06	0.001891	1.0428E-23	243.9508
TF4	0.000988	0.001748	1.2987E-18	3.5743
TF5	7.600558	63.45331	6.8427	17.2376
TF6	4.17E-16	4.36E-17	0.031252	0.0012727
TF7	0.010293	0.005973	0.00060573	0.0036346
TF8	−2857.58	-7.1E+11	−2645.1694	−3232.574
TF9	16.01883	10.44724	0.8731	2.0739
TF10	0.23103	0.280137	8.2305E-15	4.9146E-15
TF11	0.193354	0.083463	0.023527	0.034195
TF12	0.031101	8.57E-11	0.0032059	0.37982
TF13	0.002197	0.002197	0.010053	0.031895
